# Discoid Lupus Erythematosus in Genital Skin: A Case Report and Review of the Literature

**DOI:** 10.1111/cup.70145

**Published:** 2026-05-21

**Authors:** Ala' Abu‐Dayeh, Daniel Miller, Alessio Giubellino

**Affiliations:** ^1^ Department of Laboratory Medicine and Pathology University of Minnesota Minneapolis Minnesota USA; ^2^ Department of Dermatology University of Minnesota Minneapolis Minnesota USA; ^3^ Masonic Cancer Center University of Minnesota Minneapolis Minnesota USA

**Keywords:** discoid lupus erythematosus, genital dermatoses, plasmacytoid dendritic cells

## Abstract

**Background:**

Discoid lupus erythematosus (DLE) represents the most common variant of chronic cutaneous lupus erythematosus, a skin‐specific autoimmune disorder within the broader lupus erythematosus spectrum Clinically, DLE is characterized by erythematous, scaly plaques with areas of hyperpigmentation. These lesions predominantly occur on sun‐exposed areas, particularly the scalp and cheeks. Involvement of the genital skin is rare and poses a significant diagnostic challenge due to its clinical overlap with other inflammatory and infectious dermatoses. To date, only 14 cases involving genital skin have been documented in the literature, with most described in female patients and a particular tendency for the vulva to be affected.

**Case Presentation:**

We present the case of a 60‐year‐old female with a past medical history of systemic sclerosis and Raynaud's syndrome, who presented with well‐demarcated, erythematous, keratotic plaques with atrophy involving the inguinal creases and medial buttocks, associated with pruritus and recent onset of pain when sitting. The rash had been present for years. Clinical differential diagnoses included lichen sclerosus, lichen simplex chronicus, and psoriasis. Histopathologic examination revealed orthokeratosis and parakeratosis, epidermal hyperplasia, thickened basement membrane, dilated blood vessels, and perivascular inflammation. Plasmacytoid dendritic cells were present in the dermis and highlighted by CD123 immunohistochemical stain, supporting the diagnosis of DLE. Systemic evaluation was negative for systemic lupus erythematosus. The patient was started on treatment with topical corticosteroids.

**Conclusion:**

This case highlights the importance of including DLE in the differential diagnosis of chronic genital dermatoses and reinforces the value of histopathologic evaluation in such cases.

## 1 Introduction

Discoid lupus erythematosus (DLE) represents the most frequent variant of chronic cutaneous lupus erythematosus, an autoimmune condition that primarily affects the skin and belongs to the broader lupus erythematosus spectrum [1]. From a clinical perspective, DLE manifests as erythematous, scaly plaques with areas of hyperpigmentation [1]. These lesions predominantly arise on sun‐exposed areas. Genital involvement is rare and poses a significant diagnostic challenge due to its clinical overlap with other inflammatory and infectious dermatoses [2]. To date, only 14 cases involving the genital skin have been documented in the literature. Here, we present a case of genital DLE in a female patient and a summary of the cases published in the literature.

## 2 Case Presentation

A 60‐year‐old woman presented with vulvar lesions that had been present for several years, associated with pruritus and a recent onset of pain when sitting. Her medical history was notable for systemic sclerosis with positivity for antinuclear antibody (ANA), anti–Sjögren's‐syndrome‐related antigen A autoantibodies (Anti‐SS‐A), ribonucleoprotein (RNP), RNA polymerase III, and rheumatoid factor; pulmonary hypertension; gastrointestinal involvement including esophageal dilation with dysphagia and reflux; and cutaneous involvement manifested by sclerodactyly of the fingers and forearms. She also had Raynaud's phenomenon with digital pitting. Serologic evaluation showed negative anti‐double stranded DNA (anti‐dsDNA) and anti‐Smith antibodies, with normal complement (C3, C4) levels. Skin examination revealed well‐demarcated, erythematous, keratotic plaques with atrophy involving the inguinal creases and medial buttocks (Figure 1), as well as hypopigmented macules on the scalp that merged into large patches, accompanied by loss of follicular ostia and thinning throughout the scalp. The clinical differential diagnoses for the vulvar lesions included lichen sclerosus, lichen simplex chronicus, and psoriasis. Histopathologic examination demonstrated focal vacuolar interface dermatitis with scattered necrotic basal keratinocytes, orthokeratosis and parakeratosis, epidermal hyperplasia, thickened basement membrane (as highlighted by periodic acid‐Schiff [PAS]‐D staining), dermal edema, pigment incontinence, dilated blood vessels, and predominantly superficial, focally dense perivascular lymphocytic inflammation with increased plasma cells (Figure 2A–D). Periadnexal inflammation was not appreciated. Alcian blue stain highlighted interstitial dermal mucin deposition (Figure 2E). Plasmacytoid dendritic cells (pDCs), highlighted by CD123 immunostaining (Figure 2F), were present in the dermis with a mixed pattern of distribution, including focal dense clusters and scattered cells, involving the dermoepidermal junction and periadnexal areas, with rare epidermal involvement. Immunofluorescence was not performed, and systemic evaluation did not support systemic lupus erythematosus (SLE). The patient was initiated on topical corticosteroid therapy.

**FIGURE 1:**
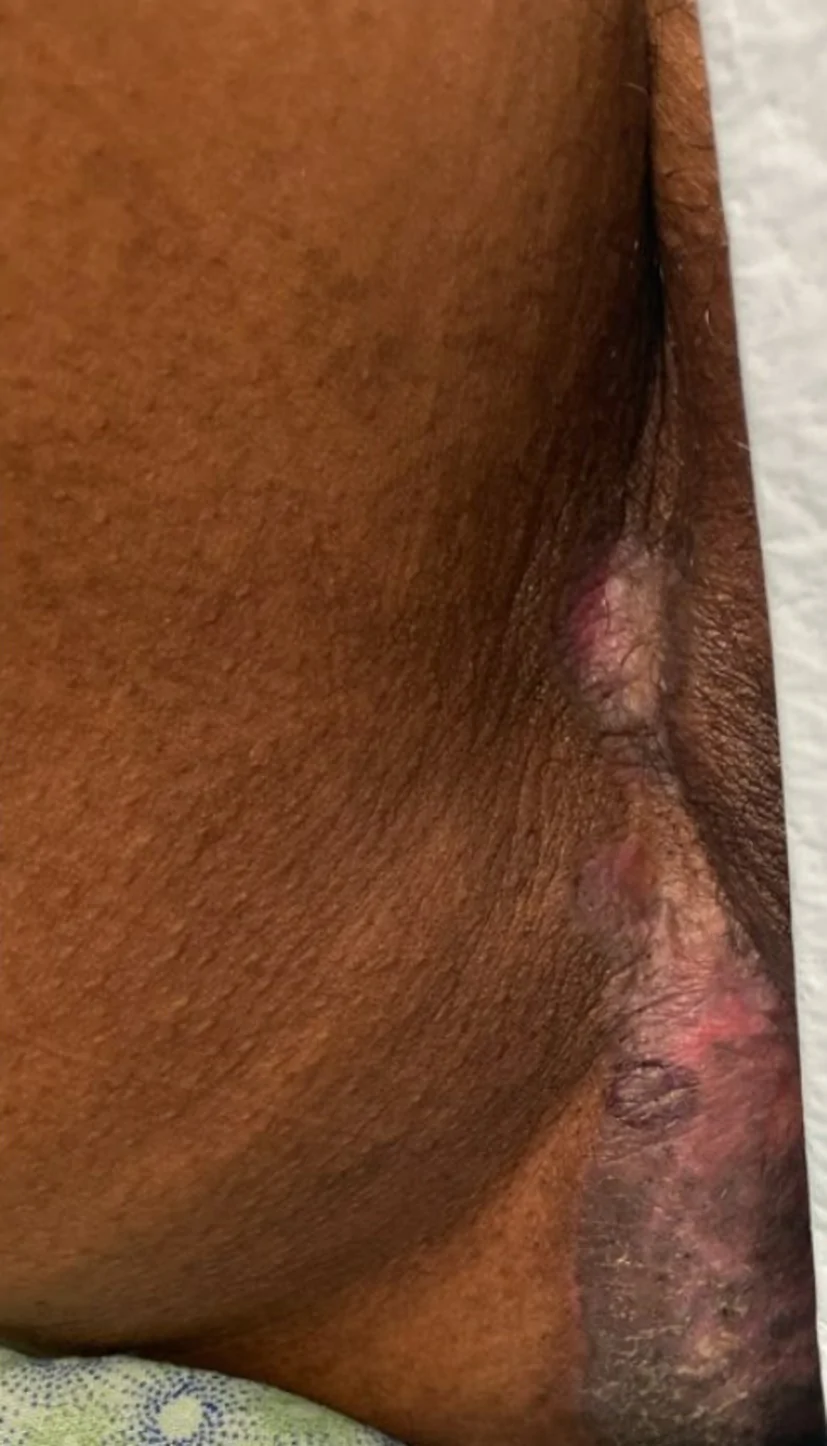
Genital discoid lupus erythematosus (DLE): Sharply defined erythematous and keratotic plaques with areas of atrophy affecting the inguinal creases and medial buttocks.

**FIGURE 2:**
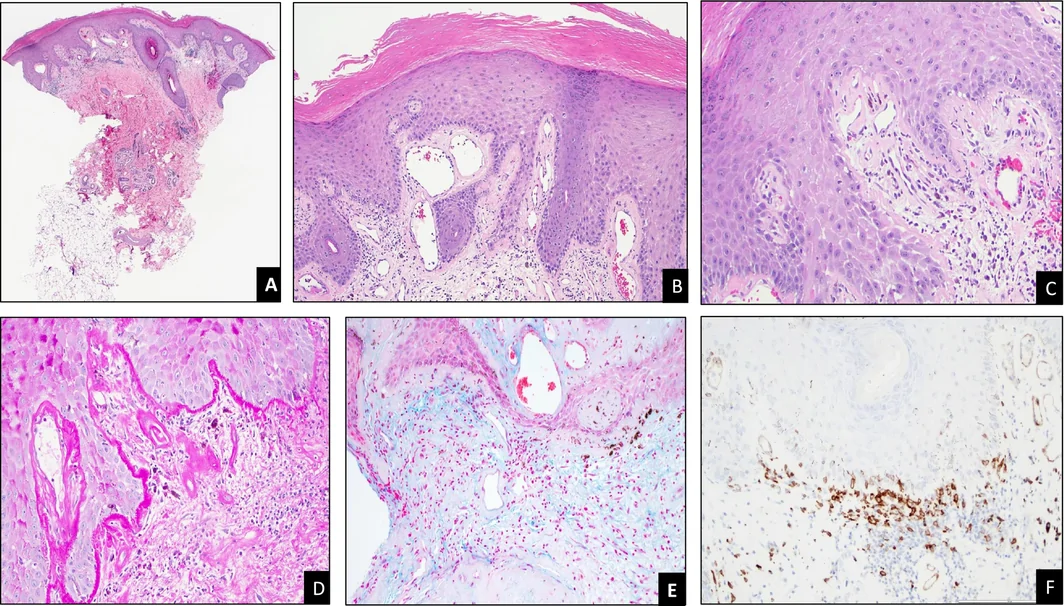
Microscopic and immunohistochemical features of genital discoid lupus erythematosus (DLE). (A) Low‐power photomicrograph showing epidermal hyperplasia with orthokeratosis (H&E stain, 20×). (B and C), Higher magnification demonstrates epidermal hyperplasia with orthokeratosis and parakeratosis, focal vacuolar interface dermatitis, thickened basement membrane, dilated blood vessels, and a perivascular lymphohistiocytic infiltrate (H&E stain, 200× and 400×, respectively). (D) Periodic acid‐Schiff (PAS) stain highlights a thickened basement membrane (200×). (E) Alcian blue stain highlights interstitial dermal mucin deposition (200×). (F) CD123 immunohistochemical stain highlights a cluster of plasmacytoid dendritic cells at the dermoepidermal junction (200×).

## 3 Discussion

DLE arises from a combination of genetic susceptibility—such as TYK2, IRF5, and CTLA4—and environmental triggers, like UV exposure, smoking, medications, and trauma. These factors provoke immune‐mediated damage through cytokine‐ and autoantibody‐driven inflammation [3]. Although genital skin is not typically UV‐exposed, local trauma or autoimmune predisposition may lead to genital involvement [4].

It typically begins as a well‐defined erythematous patch/plaque with follicular plugging, gradually expanding with peripheral inflammation and hyperpigmentation, and healing with central atrophy, scarring, and hypopigmentation [3]. Histopathologic features parallel those of extragenital DLE and include vacuolar interface change with thickening of the basement membrane; superficial and deep perivascular and periadnexal lymphohistiocytic infiltrate with increased deposition of dermal mucin; hyperkeratosis with follicular plugging; atrophic or hypertrophic epidermis with attenuation of rete ridges; dermal telangiectatic vessels [5]; and pDCs highlighted by CD123 [6]. A continuous granular band of IgG, IgA, IgM, and C3 at the basement membrane zone (BMZ) is also a characteristic on immunofluorescence studies [7]. Histopathologic differences among the various forms of lupus erythematosus are often gradual and may reflect variation in degree rather than distinct patterns. In mucosal sites such as the oral cavity, lesions may exhibit modified features due to epithelial differences, including the absence of follicular plugging [8]. Similar cytokine expression has been demonstrated in both sun‐exposed and sun‐protected lesions, supporting shared pathogenic mechanisms [9]. To our knowledge, comparable data for genital DLE are limited; however, these observations suggest that site‐specific anatomic factors may influence histopathologic presentation without representing a distinct disease process.

Genital DLE can clinically mimic several conditions, including lichen sclerosus, hypertrophic lichen planus, chronic candidiasis, psoriasis, Zoon's balanitis in males, and squamous cell carcinoma. Histopathologically, important differentials include lichen sclerosus, lichen planus, psoriasis, chronic spongiotic dermatitis, and squamous cell carcinoma [3].

Differentiating lichen sclerosus (LS) from DLE histopathologically can sometimes be challenging, as both may show interface dermatitis with lymphocytic infiltrates. LS is typically characterized by papillary dermal sclerosis, loss of elastic fibers, and a homogenized dermis [10]. An emerging consideration in this differential is the potential presence of pDCs, which are traditionally associated with lupus and other autoimmune dermatoses [11]. pDCs are a specialized dendritic cell subset with plasma cell‐like morphology that express CD4, CD123, blood‐derived dendritic cell antigen‐2 (BDCA‐2), Toll‐like receptor (TLR)7, and TLR9. Upon activation, pDCs secrete high levels of type I interferons, which play a key role in antiviral defense and in bridging innate and adaptive immune responses [11]. Interestingly, Wenzel et al. [12] identified CD123‐positive pDCs in LS lesions, suggesting that pDCs may contribute to local inflammation through type I interferon production, similar to their role in DLE.

While prominent PAS‐positive BMZ thickening is classically considered a feature of DLE [13], it can occasionally be observed in LS. LeBoit highlights that thickened BMZ may sometimes appear in LS, although it is uncommon and usually less pronounced than in DLE [14]. LS shows BMZ remodeling, with increased expression of type IV and VII collagen and reduced adhesion‐related proteins, reflecting structural alteration [15]. This imbalance suggests chronic immune‐mediated injury at the dermoepidermal junction with aberrant repair [14]. In early or nonsclerotic LS, BMZ alterations may be more evident before the development of overt dermal sclerosis, contributing to histologic overlap with other interface dermatitides [16]. The exact frequency of BMZ thickening in LS is not well established; few studies have identified this finding [17, 18].

Dilated papillary dermal blood vessels are a recognized feature of DLE but may also occur in LS. Studies have reported similar vascular changes in LS, including extragenital cases, where chronic inflammation is associated with microvascular remodeling and dilated superficial dermal capillaries as a recurrent histologic finding [19, 20].

Hypertrophic lichen planus is a key histopathologic mimic, showing pseudoepitheliomatous hyperplasia, marked hypergranulosis, and a dense band‐like lymphocytic infiltrate confined to the superficial dermis and dermoepidermal junction, sometimes with eosinophils. Direct immunofluorescence may aid distinction, with a lupus band pattern in DLE versus fibrin along the basement membrane and immunoglobulin/complement deposition in colloid bodies in hypertrophic lichen planus [21]. Other important differentials include psoriasis, which shows regular acanthosis, elongated rete ridges, confluent parakeratosis with neutrophils, and thinning of suprapapillary plates without interface change or dermal mucin [22], and chronic spongiotic dermatitis, which demonstrates spongiosis with variable acanthosis and a superficial perivascular infiltrate, often with eosinophils, and lacks basement membrane thickening.

Overlap between different dermatoses is not uncommon and can complicate diagnosis. Lupus erythematosus‐lichen planus overlap syndrome is well‐recognized and is characterized by the presence of combined histopathologic and immunologic features of both entities within a single specimen [23]. In contrast, lupus erythematosus‐LS overlap is less well described, although recent systematic review and meta‐analysis data suggest a possible association with DLE [24].

Topical corticosteroids and antimalarials remain the mainstay of treatment, while systemic agents such as immunosuppressants may be considered for refractory cases [25]. DLE is chronic and often localized; however, delayed recognition can lead to scarring, dyspareunia, and significant quality‐of‐life impairment [3]. Generalized DLE carries an estimated 28% risk of progression to SLE [26].

A total of 14 cases of genital DLE have been reported in the literature and are summarized in Table 1 [23, 27, 28, 29, 30, 31, 32, 33, 34, 35, 36]. Of these, 11 occurred in female patients, with the labia majora and minora representing the most frequently involved sites. In male patients, lesions were described on the scrotum, ventral foreskin, perineum, and inguinal region. Notably, only four patients had coexisting SLE.

**TABLE 1:** Summary of the previously published cases of genital discoid lupus erythematosus in the literature.

References	Sex	Age	Location	Lesion characteristics	Histopathologic findings	SLE?
Burge et al., 1989 [27]	F	N/A	Introitus	Ulcers	N/A	N/A
	F	N/A	Vulva	DLE	N/A	N/A
Biasi et al., 1994 [28]	F	29	Right labium minus	A shallow ulcerative erythematous lesion (5 mm) with irregular edges	Dense lymphocytic infiltrate	Yes
Bilenchi et al., 2004 [29]	F	41	Left labium majus Right labium minus (focally)	Well‐defined, slightly infiltrated, erythematous patches of hair loss, with hyperkeratotic follicles	Focal liquefaction degeneration of the basal cell layer; follicular hyperkeratosis; dilatation of small vessels; and monocytic infiltrate throughout the reticular dermis	No
Jolly et al., 2006 [30]	F	27	Left labium majus	Depressed, hypopigmented, salmon‐pink plaque	N/A	Yes
Darwish, 2008 [31]	F	54	Labia minora, anus	Ulcers	N/A	Yes
Romiti et al., 2014 [32]	F	52	Central portion of the pubic area	Keratotic and atrophic plaque (15 cm)	Interface dermatitis; hyperkeratosis and hypergranulosis; basement membrane thickening; vacuolar degeneration of the basal layer, necrotic basal keratinocytes; dilated superficial vessels; superficial and deep perivascular lymphocytic inflammation	No
	M	45	Ventral inner foreskin	Irregular patch (3 cm) consisting of ulcerations with erythema, and white keratotic area	Hyperkeratosis; vacuolar alteration of the basal layer; and superficial perivascular and interface lymphocytic infiltrates with dermal mucin	No
E. del Alcázar‐Viladomiu et al., 2018 [33]	F	65	Labia majora	Whitish, atrophic, alopecic plaques with hyperpigmented borders	Lichenoid interface dermatitis; vacuolar degeneration of the basal layer; hyperkeratosis; and mucin deposits	No
	F	35	Introitus, labia minora, and perianal region	Erosions	Focal ulceration; parakeratosis; vacuolar degeneration of the basal layer with apoptotic keratinocytes; and a chronic inflammatory infiltrate in the superficial dermis	Yes
Prenner et al., 2020 [34]	F	66	Clitoral hood and anterior labia minora	pink, depigmented, hyperkeratotic plaque with central erosions	Lichenoid interface dermatitis; epidermal hyperplasia; hyperkeratosis and hypergranulosis; follicular plugging; basement membrane zone thickening; increased dermal mucin by colloidal iron stain; and a band of CD123+ cells in the upper dermis and clusters of these cells at the dermal–epidermal junction	No
Sheriff et al., 2021 [35]	F	Mid 40s	Labia majora, labia minora, and inguinal folds	Scarred erythematous patches	Vacuolar interface dermatitis; superficial epidermal necrosis; dermal edema; and perivascular and periadnexal lymphoplasmacytic inflammation	No
Mrabat et al., 2021 [36]	M	65	Scrotum	A well‐demarcated, atrophic, erythematous, keratotic plaque (8 cm) with scales	N/A	No
Nateghi et al., 2025 [23]	M	46	Perineal and inguinal region	Erosive with scarring	Lichenoid interface dermatitis; epidermal atrophy; basement membrane thickening; lymphocytic infiltrate; and stromal mucin	No

Abbreviations: DLE, discoid lupus erythematosus; F, female; M, male; N/A, not available; SLE, systemic lupus erythematosus.

The histopathologic findings of our case closely mirror the previously reported cases, which consistently show interface dermatitis with basal layer degeneration, apoptotic keratinocytes, variable epidermal atrophy or hyperplasia, often accompanied by basement membrane thickening, dermal mucin deposition, and superficial perivascular lymphocytic inflammation. Overall, while a spectrum of changes is described, these features remain the most consistently observed pattern across cases.

Awareness of genital DLE as a diagnostic possibility is crucial for both dermatologists and dermatopathologists. Histopathologic evaluation, supported by immunohistochemical studies or direct immunofluorescence, is essential to avoid misdiagnosis and unnecessary interventions. Early recognition facilitates appropriate management and prevents chronic morbidity.

## Author Contributions

Conceptualization: all authors. Literature search and data collection: A.A.‐D. Resources: A.A.‐D. Writing – original draft preparation: A.A.‐D. Writing – review and editing: A.G. and D.M. Supervision: A.G. All authors have read and agreed to the published version of the manuscript.

## Funding

The authors have nothing to report.

## Ethics Statement

This case report was conducted in accordance with the ethical principle of the University of Minnesota.

## Consent

Informed consent was obtained from the patient involved in the study.

## Conflicts of Interest

The authors declare no conflicts of interest.

## Data Availability

The data that support the findings of this study are available from the corresponding author upon reasonable request.
